# First report of fatal disseminated microsporidiosis in two inland bearded dragons *Pogona vitticeps* in Japan

**DOI:** 10.1099/jmmcr.0.005089

**Published:** 2017-04-12

**Authors:** Kojiro Shibasaki, Toshihiro Tokiwa, Akihiro Sukegawa, Hirotaka Kondo, Kenichi Tamukai, Yumiko Haga, Kazunori Ike

**Affiliations:** ^1^​ Allieys Animal Hospital, 1-30-3 Sasazuka, Shibuya-ku, Tokyo, Japan; ^2^​ Laboratory of Veterinary Parasitology, Nippon Veterinary and Life Science University, 1-7-1 Kyonancho, Musashino, Tokyo, Japan; ^3^​ Synergy Animal General Hospital, 815 Ishigami, Kawaguchi, Saitama, Japan; ^4^​ Den-en-chofu Animal Hospital, 2-1-3 Denenchofu, Ota-ku, Tokyo, Japan

**Keywords:** *Encephalitozoon*, microsporidia, inland bearded dragons, *Pogona vitticeps*, disseminated infection, reptile

## Abstract

**Introduction.** *Encephalitozoon pogonae* is a newly described pathogen belonging to the phylum Microsporidia. In Austria and the USA, this species has been isolated from fatal and disseminated cases of captive-bred inland bearded dragons. Here, we report the case of fatal disseminated microsporidiosis caused by E. pogonae in two bearded dragons in Japan.

**Case Presentation.** The two lizards from different private households in Tokyo, Japan, had been brought to an animal hospital for examination. In both cases, the animal presented with a history of weight loss for several weeks. There were no improvements in clinical symptoms and the lizards deteriorated and finally died. Histopathological examination demonstrated necrotizing granulomatous inflammation attributed to disseminated microsporidian infection. Nucleotide sequencing of the nuclear ribosomal internal transcribed spacer region identified the microsporidian as *E. pogonae* with sequence identity of 100 %.

**Conclusion.** We report the first case, to our knowledge, of disseminated microsporidiosis caused by *E. pogonae* in inland bearded dragons in Japan. Although it is difficult to diagnose prenatally since the signs are nonspecific, the disease should be considered in the differential diagnosis of chronic infections that do not respond to antibiotics.

## Abbreviations

FNA, fine-needle aspiration; ITS, internal transcribed spacer; PAS, periodic acid–Schiff; ZN, Ziehl–Neelsen; HE, haematoxylin and eosin.

## Introduction

Microsporidia are single-celled intracellular eukaryotes. They have recently been reclassified from protozoa to fungi and can infect a wide range of vertebrate hosts, including humans, and cause microsporidiosis [1, 2]. In reptiles, although few reports on cases of microsporidiosis have been published, at least three genera of microsporidia have been reported: *Encephalitozoon*, *Heterosporis* and *Pleistophora* (syn. *Glugea*) [3]. In recent years, microsporidiosis of inland bearded dragons has been reported in Austria and the USA and *Encephalitozoon pogonae* has been described from a fatal case of disseminated infection [4–7]. Here, we report the first case, to our knowledge, of fatal disseminated microsporidiosis caused by *E. pogonae* in two bearded dragons in Japan.

## Case Report

**Fig. 1.**
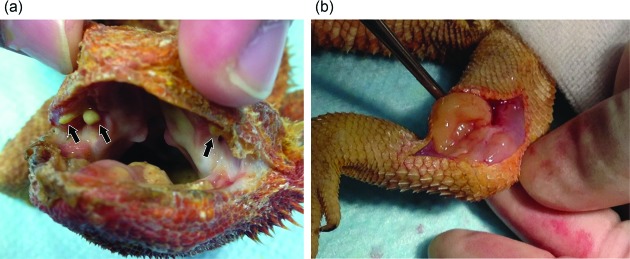
Lesions associated with microsporidiosis in inland bearded dragon (case 1). (a) Soft masses (arrows) are located in the oral cavity. (b) Soft and fat-like masses are located in the swollen left femur.

**Fig. 2.**
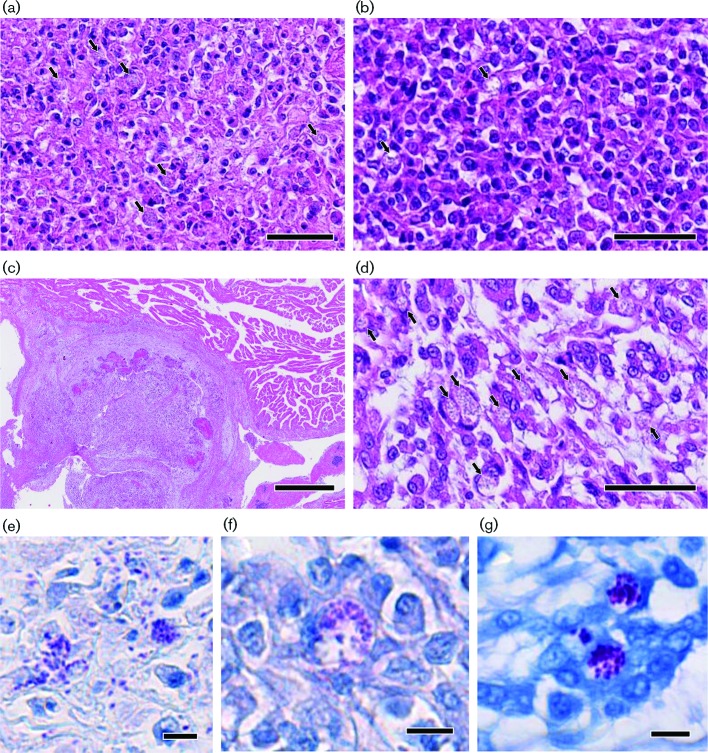
Disseminated microsporidiosis associated with *Encephalitozoon pogonae* on inland bearded dragons. (a) Intraoral mass showing necrosis and microsporidian spores in a parasitophorous vacuole (arrows). HE staining. Bar, 50 µm. (b), Tissue of periarticular lesion consists mainly of lymphocytes. Arrows indicate a group of spores within macrophages. HE staining. Bar, 50 µm. (c, d) Nodular lesion found in myocardium showing granulomatous inflammation and many microsporidian spores in a parasitophorous vacuole. HE staining. Bars, 1 mm (c) and 50 µm (d). (e–g) High magnification of spores found in the tissue of the mass in the oral cavity (e) and periarticular (f) and myocardial (g) regions showed similar morphological characteristics. ZN staining. Bars, 10 µm.

The first animal (case 1) was a female inland bearded dragon aged 2 years and 10 months old, weighing 228 g, which presented with a history of weight loss and head tremor. These symptoms had also been observed one year previously and diagnosed as resulting from hypocalcaemia in a different hospital. On physical examination, a purulent lesion was found in the oral cavity, so enrofloxacin (5 mg kg^−1^, p.o., s.i.d.) was administered for 14 days. On day 35, an intraoral mass of 7 mm in diameter was found on the upper jaw. On day 75, fine-needle aspiration (FNA) cytology of the mass showed the presence of numerous inflammatory cells and *Pseudomonas aeruginosa* was isolated by bacterial culture. On day 84, amikacin (20 mg kg^−1^, subcutaneous,weekly) and sulbactam–cefoperazone (10 mg kg^−1^, subcutaneous, weekly) were administered. On day 98, tebipenem pivoxil (5 mg kg^−1^, orally, twice daily) was administered. On day 115, the mass was surgically excised because it did not respond to medical therapy. Histologically, the mass was composed of necrogranulomatous inflammation surrounded by small lymphoid cells. Microorganisms that were positive for periodic acid–Schiff (PAS) reaction and Ziehl–Neelsen (ZN) staining, were not observed in the tissue. On day 120, swelling was observed in the soft tissue of the left knee. The animal survived for 140 days. On necropsy, the animal was emaciated, with bilateral renal atrophy, pericardial effusion and recurrence of the intraoral mass (Fig. 1a). A soft and fatty mass was observed in the left periarticular region (Fig. 1b). Tissue samples of kidney, lung, liver, intestine, pancreas, heart, spleen, ovary and lesions of the oral cavity and knee joint were fixed in 10 % neutral buffered formalin for histological examination. Haematoxylin and eosin (HE)-stained sections of the intraoral mass (Fig. 2a), kidney and heart (Fig. 2c, d) showed necrogranulomatous inflammation. Similar foci were observed in the tissues of lung and periarticular mass (Fig. 2b). PAS and ZN staining revealed clusters of small and oval spores that filled and distended the cytoplasm of host cells in the tissue of the kidney, lung, heart (Fig. 2g), and the lesions of the oral cavity (Fig. 2e) and knee joint (Fig. 2f). These spores were ovoid in shape and measured about 1–2 µm. Liver showed lipidosis. Lesions and spores were not observed in the other tissues.

The second animal (case 2) was a male inland bearded dragon aged 1 year and 8 months old, weighing 278 g. It had a history of weight loss, which was observed two months previously, but the animal had a healthy appetite. On physical examination, the animal was emaciated and mildly dehydrated. Faecal examination revealed no helminth eggs. Whole-body lateral and ventrodorsal radiographs revealed a mass in the left lung area. The animal was administered enrofloxacin (5 mg kg^−1^, subcutaneous), prednisolone (1 mg kg^−1^, subcutaneous) and scopolamine butylbromide (2 mg kg^−1^, subcutaneous). On day 3, the animal was lethargic and received enfloxacin (10 mg kg^−1^, subcutaneous). On day 4, the animal was found dead, and its weight was 270 g. On necropsy, severe emaciation and nodules in the left lung were observed. Tissue samples of lung, kidney, adrenal glands, liver, stomach, intestine, heart and spleen were fixed in 10 % neutral buffered formalin for histological examination. HE-stained sections of the lung showed mild infiltration of lymphocytes, plasma cells and histocytes in the interstitial and peribronchial tissues. The renal and adrenal parenchyma was expanded and replaced by multifocal to coalescing necrogranulomatous inflammation with intrahistiocytic clusters of small and oval spores, which were positive for PAS and ZN staining. Similar foci were observed in tissues of the stomach and intestines. No significant lesions were observed in the heart and splenic tissues.

## Investigations

For molecular analyses, total genomic DNA was extracted from formalin-fixed and paraffin-embedded specimens. For case 1, 5 µm kidney sections adhered to three glass slides were picked up by plastic pipette tips. For case 2, seven 5 µm sections of kidney were newly sectioned. These specimens were collected in 1.5 ml tubes for each case and extracted with DEXPAT Easy (TaKaRa), in accordance with the manufacturer's instructions. The nuclear ribosomal DNA, including the entire internal transcribed spacer (ITS), region was amplified using oligonucleotides EP1279F (5′-GAA CCA GCA GCA GGA TCA GT-3′) and EP1410R (5′-ATA TCG TCG TCC TCC ACG TC-3′) as a primer pair, which were designed based on ribosomal DNA sequence alignments of *E. pogonae* (GenBank/DDBJ/EMBL database accession number KR998311), *Encephalitozoon* *cuniculi* (AJ005581) and *Encephalitozoon hellem* (AF177920, AF272836). PCR was carried out in 20 µl of reaction mixture (Ex Taq; Takara) containing 0.2 µl Ex Taq (5 units µl^−1^), 2.0 µl of 10×Ex Taq Buffer, 1.6 µl dNTP mixture (2.5 mM each), 0.2 µl of each primer (50 µM), 1 µltemplate DNA, and 14.8 µl pure water. The amplification schedule consisted of initial denaturation at 94 °C for 2 min; followed by 40 cycles at 94 °C for 30 s, 53 °C for 30 s, and 72 °C for 30 min; and then a final extension step at 72 °C for 2 min. The amplified DNA was applied to 2 % agarose gels, electrophoresed and visualized under an LED transilluminator. PCR products were purified with ExoSAP-IT (Affymetrix) and both strands were sequenced with the same primers as used for the PCR. The obtained sequences were analysed with mega version 7.0 [8]. The results indicated that the specific DNA fragment with an expected size of 132 bp was successfully amplified and sequenced from both samples. These sequences were 100 % identical and deposited in the DDBJ database under the accession number LC214885. Analysis using the nucleotide basic local alignment search tool (www.ncbi.nlm.nih.gov/Blast.cgi) demonstrated that the obtained ITS sequence had 100 % identity (query cover 100 %) with *E. pogonae* (KR998311) isolated from the small intestine of an inland bearded dragon from the USA [7] and an uncultured clone of the genus *Encephalitozoon* (JQ682643) isolated from small intestinal tissue of an inland bearded dragon from the USA [5].

## Discussion

Infection with microsporidian organisms has been reported in a range of reptiles, and the majority of these reports are on infection of Squamata such as lizards and snakes [3, 7]. In the suborder Iguania, *Encephalitozoon lacertae* was isolated from the common wall lizard, *Podacris muralis* [9], and was found to be morphologically similar to *E. lacertae* isolated from the African skink, *Mabuya perrotetii* [10]. In inland bearded dragons, several cases of necrotizing or granulomatous inflammation associated with disseminated intracellular microsporidia infection have been reported. Jacobson *et al*. [4] reported three cases in the USA and diagnosed them as microsporidiosis by optical and transmission microscopy observation. In addition, Richter *et al*. [5] reported two cases of disseminated granulomatous diseases due to a novel genotype of *E. cuniculi* in Austria. Schilliger *et al*. [6] also reported *E. cuniculi*-like microsporidia in an inland bearded dragon in the EU. Sokolova *et al*. [7] described *E. pogonae* as a novel species from a study of isolates from inland bearded dragons with disseminated granulomatous diseases in the USA, and the species found in bearded dragons in Austria was later classified as *E. pogoinae* as well. In this study, intracellular microorganisms were found in two inland bearded dragons with similar pathological lesions. The spores found in the present study present similar shape and size to those of other species of the genus *Encephalitozoon* parasitizing reptiles, for example, *E. cuniculi*, *E. hellem* and *E. intestinalis*. These can be distinguished morphologically from another reptilian microsporidium, *E. lacerate,* by their smaller size and less elongated shape [7]. Analysis based of the ITS region sequence of the detected microorganisms revealed the divergence of *E. pogonae* from other reptilian pathogens. To the author's knowledge, this is the first report of *E. pogonae* in Asia.

The clinical sign shared by the two animals described here is weight loss. The course was chronic and the animals showed anorexia, lethargy and finally death, which were similar to the cases reported in Austria and USA [4–7]. Although it is difficult to diagnose prenatally since these signs are nonspecific, microsporidiosis should be considered in the differential diagnosis of chronic systemic infections that do not respond to antibiotics. In the present study, numerous spores were found in kidney and lung in both cases and also in the intestinal tract. This indicates that spores are excreted in the intestinal and/or urinary tracts and cause horizontal transmission through the faecal-oral route. In case 1, spores were not found in the FNA specimen of the intraoral mass on day 115, but they were found on day 140. During this period, it is unlikely that the animal contacted the source of infection. Intriguingly, the present two animals were purchased at the same facility in Tokyo and kept in different private households. This raises the possibility that these animals had already been infected with *E. pogonae* in the facility but had not demonstrated clinical signs.

Phylogenetic analysis using ribosomal DNA sequences revealed a close relationship between *E. pogonae* isolated from inland bearded dragons and *E. cuniculi* isolated from human, fox, mouse, rabbit and dog [7]. Furthermore, the sequence diversity of the non-coding regions of genomic DNA, such as the ITS region of the ribosomal RNA gene, has already been demonstrated to include polymorphisms that allow differentiation among individual strains of the genus *Encephalitozoon.* In the case of *E. cuniculi,* four different strains have so far been differentiated by the tetranucleotide repeat (5′-GTTT-3′) in the ITS sequence [11–13]. Although there seems to be a host preference of strains it does not seem to be strict [2]. For *E. hellem,* small deletions or insertions and some point mutations differentiate the three strains [2, 14]. In the present study, although the number of specimens was small, genetic diversity was not found among *E. pogonae* isolates from inland bearded dragons in Japan and isolates from the USA and possibly *E. pogonae* isolated from Austria. These results reinforce the hypothesis of a clonal distribution of *E. pogonae* in inland bearded dragons worldwide.

At least eight species have been recognised in the genus *Pogona,* all of which are medium in size, diurnal and omnivorous, feeding on plants, small vertebrates and insects [15]. These species are native to the arid and semiarid habitats of inland Australia. Within the genus, inland bearded dragons are the most common captively bred bearded dragon species. Since the 1960s, the export of all wildlife, including bearded dragons, out of Australia has been illegal [16]. This means that most bearded dragons found outside Australia today are captively bred and probably the descendants of animals smuggled out of the country since the 1970s. In Japan, this species has become popular in the reptile trade and numerous individuals for breeding were imported from USA, Germany and Thailand. We believe that the international reptile pet industry plays a major role in the intercontinental spread of alien pathogens such as *E. pogonae*, characterised by low levels of sequence diversity. In conclusion, this is, to our knowledge, the first case of disseminated microsporidiosis caused by *E. pogonae* in Japan. We expect that further information on the prevalence of *E. pogonae* infection in asymptomatic inland bearded dragons and other reptiles kept in commercial facilities will yield critical insights that will help to clarify the transmission route of this disease.

## References

[1] Mathis A, Weber R, Deplazes P (2005). Zoonotic potential of the microsporidia. Clin Microbiol Rev.

[2] Hinney B, Sak B, Joachim A, Kváč M (2016). More than a rabbit's tale – *Encephalitozoon* spp. in wild mammals and birds. Int J Parasitol.

[3] Vergneau-Grosset C, Larrat S (2016). Microsporidiosis in vertebrate companion exotic animals. J Fungi.

[4] Jacobson ER, Green DE, Undeen AH, Cranfield M, Vaughn KL (1998). Systemic microsporidiosis in inland bearded dragons (*Pogona vitticeps*). J Zoo Wildl Med.

[5] Richter B, Csokai J, Graner I, Eisenberg T, Pantchev N (2013). Encephalitozoonosis in two inland bearded dragons (*Pogona vitticeps*). J Comp Pathol.

[6] Schilliger L, Mentré V, Marschang RE, Nicolier A, Richter B (2016). Triple infection with agamid adenovirus 1, *Encephaliton cuniculi*-like microsporidium and enteric coccidia in a bearded dragon (*Pogona vitticeps*). Tierarztl Prax Ausg K Kleintiere Heimtiere.

[7] Sokolova YY, Sakaguchi K, Paulsen DB (2016). Establishing a new species *Encephalitozoon pogonae* for the microsporidian parasite of inland bearded dragon *Pogona*
*vitticeps* Ahl 1927 (Reptilia, Squamata, Agamidae). J Eukaryot Microbiol.

[8] Kumar S, Stecher G, Tamura K (2016). MEGA7: molecular evolutionary genetics analysis version 7.0 for bigger datasets. Mol Biol Evol.

[9] Canning EU, Canning EU (1981). *Encephalitozoon lacerate* n. sp., a microsporidian parasite of the lizard *Podarcis muralis*. Parasitological Topics.

[10] Koudela B, Didier ES, Rogers LB, Modrý D, Kucerová S (1998). Intestinal microsporidiosis in African skink *Mabuya perrotetii*. Folia Parasitol.

[11] Xiao L, Li L, Moura H, Sulaiman I, Lal AA (2001). Genotyping *Encephalitozoon hellem* isolates by analysis of the polar tube protein gene. J Clin Microbiol.

[12] Talabani H, Sarfati C, Pillebout E, van Gool T, Derouin F (2010). Disseminated infection with a new genovar of *Encephalitozoon cuniculi* in a renal transplant recipient. J Clin Microbiol.

[13] Galván A, Magnet A, Izquierdo F, Fenoy S, Henriques-Gil N (2013). Variability in minimal genomes: analysis of tandem repeats in the microsporidia *Encephalitozoon intestinalis*. Infect Genet Evol.

[14] Xiao L, Li L, Visvesvara GS, Moura H, Didier ES (2001). Genotyping *Encephalitozoon cuniculi* by multilocus analyses of genes with repetitive sequences. J Clin Microbiol.

[15] Uetz P (2010). The original descriptions of reptiles. Zootaxa.

[16] Hose RT, Hoser RT (1993). Real smuggling. Smuggled: The Underground Trade in Australia's Wildlife.

